# Kombucha Fermentation With Dried Starter Cultures: A Strategy for Microbial Stabilization via Spray and Freeze Drying

**DOI:** 10.1111/1750-3841.70474

**Published:** 2025-08-11

**Authors:** Alliny Samara Lopes de Lima, Ana Terra de Medeiros Felipe, Maria Eduarda de Souza da Cruz, Luiz da Silva Ferreira Junior, Francisco Canindé de Sousa Junior, Fábio Gonçalves Macêdo de Medeiros, Márcia Regina da Silva Pedrini

**Affiliations:** ^1^ Department of Chemical Engineering, Laboratory of Bioprocess Technology Center, Federal University of Rio Grande do Norte Natal Brazil; ^2^ Department of Chemical Engineering, Laboratory of Quality Control Technology Center, Federal University of Rio Grande do Norte Natal Brazil; ^3^ Department of Pharmacy, Laboratory of Bromatology Technology Center, Federal University of Rio Grande do Norte Natal Brazil

**Keywords:** encapsulation, fermentation, SCOBY

## Abstract

Kombucha production relies on complex microbial consortia, which often vary across batches, posing challenges for fermentation reproducibility, product standardization, and industrial scalability. This study explored the effects of spray drying (SD) and freeze drying (FD) on the viability and metabolic activity of kombucha starter cultures, using maltodextrin (MD) and gum Arabic (GA) as drying carriers. The dried starter cultures exhibited favorable physicochemical properties, including low moisture content (2.22 – 3.62%), reduced water activity (0.135 – 0.230), and high solubility (> 90%). Fermentation performance varied according to the drying method and carrier used. Acetic acid concentrations in beverages fermented with dried cultures ranged from 1.49 to 2.67 g/L, with the lowest values in the MD‐SD treatment, suggesting thermal damage to microbial cells. In contrast, GA effectively protected microorganisms, as indicated by comparable acid production and minimal reductions (<1 log CFU/mL) in viable cell counts between GA‐SD and GA‐FD treatments. Ethanol concentrations ranged from 1.84 to 2.67 g/L, with no significant differences among treatments. Total phenolic content ranged from 271.32 to 391.47 mg GAE/L, with higher values observed in beverages fermented with freeze‐dried cultures, corresponding to enhanced antioxidant activity. These findings confirm the feasibility of producing kombucha starter cultures in a dried form, offering a potential avenue for microbial stabilization and industrial process standardization.

**Practical Applications:**

This research demonstrates that kombucha starter cultures can be effectively dried using spray‐drying or freeze‐drying techniques, preserving their microbial viability and fermentation capabilities. These stable, dry cultures can be easily stored, transported, and reactivated, offering a practical solution for the industrial production of kombucha and other fermented beverages.

## 1 Introduction

Kombucha is a fermented beverage that has gained increased attention in the functional beverage sector due to its health‐relevant properties and sensory acceptance (Ariff et al. 2023; Giuffrè and Giuffrè 2024). Kombucha production results in a low‐calorie, carbonated beverage characterized by a slightly sweet and acidic taste, offering a healthy alternative to traditional soft drinks and presenting a promising market with significant growth potential. The global kombucha market is projected to reach between US$3.5 bi—US$5 bi by 2025 (De Melo et al. 2024). The functional properties of kombucha are mainly associated with antioxidant, anti‐inflammatory, and antimicrobial activities (Cardoso et al. 2020; Içen et al. 2023) These properties are attributed to bioactive compounds derived from tea infusions and metabolites produced during fermentation, such as organic acids, vitamins, and polyphenols (Bortolomedi et al. 2022; Liang et al. 2024).

The beverage is typically produced through the fermentation of green or black tea (*Camellia sinensis* leaves) infusions with sucrose by a symbiotic culture of bacteria and yeast (SCOBY). This process results in a tea‐like fermented liquid and a floating cellulose film (biofilm), both of which can be utilized as starter cultures in subsequent fermentations, either individually or in combination (Arıkan et al. 2020; Savary et al. 2021). Kombucha fermentation is conducted by a symbiotic complex microbial consortium with variable composition, consisting of acetic acid bacteria (e.g., *Acetobacter* and *Komagataeibacter*) and yeasts (e.g., *Zygosaccharomyces* and *Dekkera/Brettanomyces*), while lactic acid bacteria (e.g., *Lactobacillus* and *Lactococcus*) may or may not be present (Bishop et al. 2022; Fabricio et al. 2022; Villarreal‐Soto et al. 2020). This non‐standardized microbial community can undergo mutations and modifications along the propagation process, which can result in variations of kombucha chemical and sensory profiles, leading to standardization challenges for the beverage production (Sogin and Worobo 2024).

Drying techniques are widely employed for encapsulating yeast and probiotic cultures, providing significant advantages such as enhanced strain stability, maintenance of cell viability, and reduced costs associated with storage and transportation (Kandasamy and Naveen 2022; Misra et al. 2023). Among these methods, freeze‐drying is widely applied due to its efficacy in preserving heat‐sensitive compounds, yielding consistent results in the stabilization of dried microbial strains (Benkirane et al. 2024). A cost‐effective alternative to freezing‐drying is spray drying, characterized by its continuous operation and high processing efficiency (Samborska et al. 2022; Demircan et al. 2023). Studies have successfully demonstrated the encapsulation of heat‐sensitive compounds, including microbial cells and bioactive compounds, within polymeric matrices by spray drying (De Carvalho et al. 2024; Hoskin et al. 2023; Moraes et al. 2024). Encapsulation plays an important role in avoiding thermosensitive compound damage, thereby improving drying efficiency and enhancing storage stability (Rezende et al. 2018; Zanoelo et al. 2024). Maltodextrin and gum Arabic are common wall materials used in drying encapsulation, with maltodextrin valued for its high solubility and low cost, while gum Arabic is recognized for its emulsifying and film‐forming properties (Benkirane et al. 2024; Laureanti et al. 2023; Obradović et al. 2022).

Despite the growing interest in kombucha, challenges related to process standardization and starter culture stabilization are still to be resolved. Drying techniques offer potential solutions, but their impact on the microbial viability and bioactivity of kombucha is not fully understood. Therefore, the objective of this study was to evaluate the feasibility of using spray drying and freeze drying to produce stable kombucha starter cultures and assess their impact on fermentation performance. The dried cultures were characterized based on their physicochemical properties, microbial viability, and fermentative performance, with monitoring of parameters such as viable cell counts, organic acid and ethanol production, and final product parameters including total phenolic content (TPC) and antioxidant activity. This study aimed to contribute to the development of scalable and standardized starter cultures for potential application in industrial kombucha production.

## 2 Materials and Methods

### 2.1 Materials

The kombucha starter culture, consisting of fermented liquid and cellulose biofilm, was obtained from a local company (Natal, RN, Brazil) and kept at 4°C until use. Green tea was purchased from Vemat (Xanxerê, SC, Brazil) and stored in airtight containers, protected from light, at room temperature until use. White granulated sugar was obtained from Ecoçúcar (Maringá, PR, Brazil). Maltodextrin was obtained from Nutylac (Sorocaba, SP, Brazil), and gum Arabic from Nexira (Rouen, Normandy, France). Ethanol, methanol, sodium carbonate (NaHCO_3_), and gallic acid were purchased from Êxodo Científica (Sumaré, SP, Brazil). The Folin‐Ciocalteu reagent, 2,2′‐azino‐bis (3‐ethylbenzothiazoline‐6‐sulfonic acid) (ABTS), (±)‐6‐hydroxy‐2,5,7,8‐tetramethylchroman‐2‐carboxylic acid (Trolox), 2,2‐diphenyl‐1‐picrylhydrazyl (DPPH) were purchased from Sigma‐Aldrich (St. Louis, Missouri, USA). Plate count agar (PCA) was obtained from Himedia (Mumbai, Maharashtra, India); buffered peptone water and potato dextrose agar (PDA) from Merck (Darmstadt, Hesse, Germany); yeast extract and bacteriological agar from KASVI (São José dos Pinhais, PR, Brazil); calcium carbonate from Synth (Diadema, SP, Brazil); cycloheximide from INLAB (Vila Campestre, SP, Brazil); phosphoric acid and glucose from Vetec (RJ, Brazil).

### 2.2 Kombucha Fermentation From Liquid Starter Culture

Green tea infusion (0.6% w/v) was prepared by steeping dried leaves of *Camellia sinensis* in hot water (85 ± 5°C) for 10 min, followed by filtration and the addition of 8% w/v of white granulated sugar. After cooling to room temperature, 20% v/v of fermented kombucha liquid (pH = 3.8) was added as a starter culture. A total of 1.6 L of kombucha was added to each sterilized glass jar (22 cm × 12 cm). Fermentation was performed at room temperature (28 ± 4°C) under aerobic conditions, covered with paper towels, for 14 days. After fermentation, the kombucha cellulosic biofilm formed on the surface of the containers was separated from the kombucha fermented liquid under aseptic conditions using filtration. Both the biofilm and the liquid were transferred to sterile containers for microbial suspension preparation and subsequent drying processes.

### 2.3 Drying Processes

The drying of fermented kombucha to obtain a dried starter culture was evaluated through spray drying (SD) and freeze drying (FD) using maltodextrin (MD) and gum Arabic (GA) as drying carriers for a total of four experimental treatments: MD‐SD (fermented kombucha with maltodextrin, spray dried); MD‐FD (fermented kombucha with maltodextrin, freeze dried); GA‐SD (fermented kombucha with gum Arabic, spray dried); and GA‐FD (fermented kombucha with gum Arabic, freeze dried). Feed solutions were prepared by aseptically mixing (LQI‐08, Vitalex, Brasil) 600 g of cellulosic biofilm (wet basis; moisture content = 89.57 ± 0.97%) and 3 L of kombucha fermented liquid, both obtained as described in Section 2.2, for 5 min. The final kombucha suspension (fermented liquid and biofilm) reached a total solids content of 7.85 ± 1.5%. After, drying carriers were added at 15% w/v to kombucha suspensions, and solutions were mechanically stirred at room temperature for 5 min until complete carrier dissolution. Freeze‐dried samples (MD‐FD and GA‐FD) were frozen at ‐80°C for 24 h (CL 200–86 V, Coldlab, SP, Brazil) and freeze‐dried (L101, LIOBRAS, Brazil) for 72 h at ‐ 55°C, 13 kPa. Spray‐dried samples (MD‐SD and GA‐SD) were obtained in a lab‐scale spray dryer (MSD 1.0, LabMaq, SP, Brazil) using a 1.0 mm nozzle at a 4.2 mL/min feed flow rate, controlled by a peristaltic pump and kept under constant magnetic stirring at 30°C, and using air at 45 L/min in co‐current flow. A total of 500 g of each sample was used as feed for the spray‐drying process. Drying was performed with an inlet air temperature of 110°C and an outlet temperature of 70 ± 2°C, a condition selected based on preliminary tests (data not shown) and consistent with spray‐drying conditions commonly applied in probiotic formulations (Misra et al. 2023; Ngamekaue et al. 2024). All drying experiments were conducted in triplicate, and dried kombucha powders were stored in airtight bags, protected from light, and frozen at ‐ 20°C until further analysis.

### 2.4 Characterization of Dried Kombucha Starter Cultures

#### 2.4.1 Moisture Content, Water Activity, and pH

The moisture content on a wet basis was determined gravimetrically at 105°C with 3 g of dried kombucha powders (AOAC 2023). Water activity (Aw) was determined by direct measurement in a water activity analyzer (S3TE, Aqualab, USA) at room temperature (28 ± 2°C). The pH of 10% w/v dispersions of dried kombucha powder samples in distilled water was determined using a PHS‐3E pH meter (IONLAB, PR, Brazil) (Moraes et al. 2024).

#### 2.4.2 Hygroscopicity and Solubility

Hygroscopicity was measured by adding a 0.5 g of the sample to a desiccator containing saturated NaCl solution (RH 75.3%). After 7 days, the samples were weighed, with hygroscopicity expressed in grams of adsorbed water per 100 g of powder (g H_2_O/100 g sample). Solubility was determined by adding 0.5 g sample of dried powder in 50 mL of distilled water (1% w/v). The mixture was vortexed for 5 min and centrifuged at 4000 rpm for 5 min. The supernatant (25 mL) was dried in an oven at 105°C for 5 h. Solubility was calculated as the ratio between the total solids of dried supernatant and the total solids of the initial powder sample, expressed as a percentage (%) (Silva et al. 2024).

#### 2.4.3 Scanning Electron Microscopy (SEM) and X‐Ray Diffraction (XRD)

Kombucha powders were sieved through a 400‐mesh screen. The morphology of the powders was examined using a scanning electron microscope (SEM TM3000, Hitachi, USA) with Au metallization (Misra et al. 2023). The samples were analyzed at 5 kV and magnifications of 500x, 1000x, and 2000x. The microstructure of particles was evaluated using a D2 PHASER diffractometer (Bruker, Massachusetts, USA) over a 2θ range between 5° to 70° and an angular pitch of 0.02°.

### 2.5 Kombucha Fermentation From Dried Starter Culture

**FIGURE 1:**
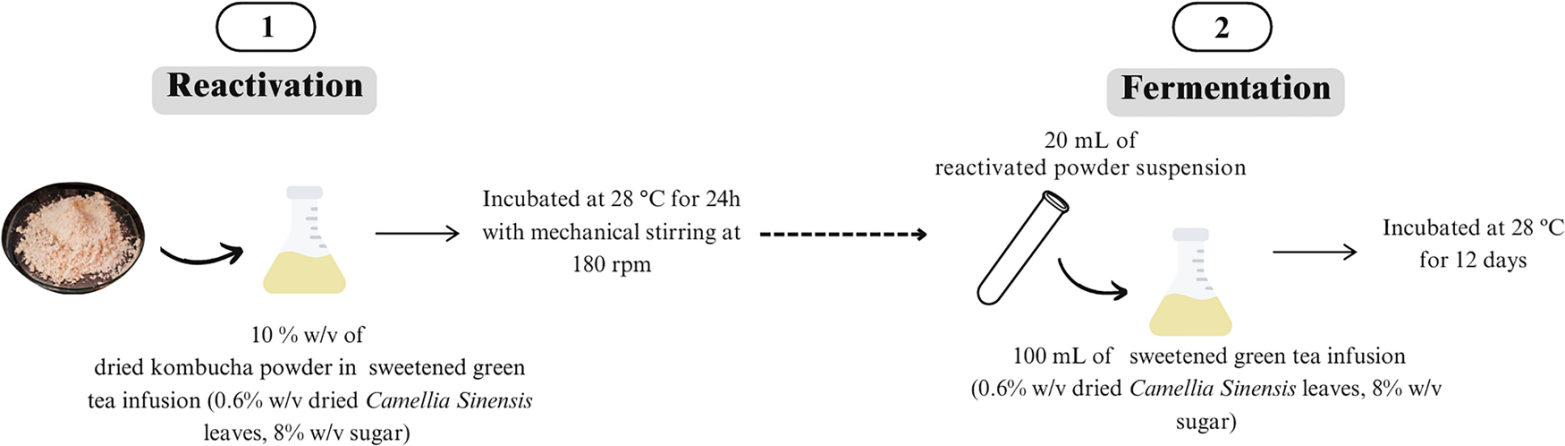
Schematic representation of the two‐step process (reactivation followed by fermentation) for kombucha production using dried starter cultures.

Kombucha fermentation using dried starter culture was performed in a two‐step process (Figure 1). First, the dried starter cultures were reactivated at a concentration of 10% w/v in sweetened green tea infusion (0.6% w/v dried *Camellia sinensis* leaves steeped in hot water (85 ± 5°C) for 10 min, added to 8% w/v granulated sugar, pH adjusted to 3.8 with 0.05 M phosphoric acid, and filtered through a 0.22 µm nylon membrane) (Fabricio et al. 2022). The suspensions were incubated in 50 mL Erlenmeyer flasks covered with paper towels for 24 h at 28°C, 180 rpm (TE‐422, Tecnal, SP, Brazil). After incubation, fresh sweetened green tea infusion was inoculated (20% v/v inoculum rate) with the reactivated starter culture suspensions and incubated (TE‐422, Tecnal, SP, Brazil) in flasks covered with paper towels under static conditions for 12 days at 28°C. Samples were collected after 0 (initial condition), 3, 6, 9, and 12 days of fermentation, filtered through a 0.22 µm membrane filter, and stored frozen until analysis. A control fermentation was performed under identical conditions using a fresh kombucha liquid starter culture fermented for 12 days, the same culture used for preparing the kombucha suspension, as detailed in Section 2.2.

### 2.6 Kombucha Fermentation Characterization

#### 2.6.1 Determination of pH, Acetic Acid, and Ethanol Content

The pH values of the kombucha beverages fermented with dried starter culture were measured using a PHS‐3E pH meter (IONLAB, PR, Brazil) previously calibrated. The concentrations of acetic acid and ethanol were quantified using high‐performance liquid chromatography (HPLC) on a Shimadzu LC10ADvp liquid chromatography (Shimadzu, Japan) equipped with a refractive index detector. Samples were filtered through 0.22 µm pore size membranes, and 20 µL was injected into a Shim‐Pack SCR‐101H column (7.9 mm i.d. × 300 mm, 10 µm) (Shimadzu, Japan) at 65°C. Sulfuric acid solution (0.005 M) was used as the mobile phase at a flow rate of 0.6 mL/min (Ribeiro et al. 2019). Analyses were performed on samples collected at 0, 3, 6, 9, and 12 days of fermentation.

#### 2.6.2 Enumeration of Viable Cells

Viable cell counts were estimated using the colony‐forming unit (CFU) method. For total aerobic bacteria, samples were plated onto standard Plate Count Agar (PCA) at incubated for 72 h at 30°C. Acetic acid bacteria were quantified using GYC medium (10 g/L yeast extract, 50 g/L glucose, 5 g/L calcium carbonate, 20 g/L agar; pH: 6.8) supplemented with 100 mg/L of cycloheximide and incubated at 30°C for 48 h (Kim et al. 2019). Yeast counts were determined using potato dextrose agar supplemented with 100 mg/mL chloramphenicol, incubated at 25°C for 5 days (Grassi et al. 2022). All analyses were performed in triplicate, and the results were expressed as colony‐forming units per milliliter of sample (CFU/mL). Analysis were performed on fermentation days 0, 6, and 12.

#### 2.6.3 TPC

The TPC of kombucha samples was quantified using a microplate Folin‐Ciocalteu assay (Singleton et al. 1999). Briefly, 25 µL of filtered sample (0.45 µm, Sartorius Stedim) was mixed with 50 µL of distilled water, 25 µL of 1 M Folin‐Ciocalteu reagent (1:1 dilution), and 100 µL of 7.5% (w/v) sodium bicarbonate solution in a 96‐well plate. The plates were incubated for 90 min at room temperature in the dark, and the absorbance was measured at 765 nm using a UVM 340 microplate reader (Biochrom, Cambridge, England). A standard curve was constructed using varying concentrations of gallic acid (6.5‐250 mg/L), and the results were expressed as milligrams of gallic acid equivalents per liter of sample (mg GAE/L).

#### 2.6.4 Antioxidant Activity

The antioxidant capacity of the kombucha samples was assessed using the 2,2‐diphenyl‐1‐picrylhydrazyl (DPPH) and 2,2'‐azino‐bis (3‐ethylbenzothiazoline‐6‐sulfonic acid) (ABTS) radical scavenging assays. The DPPH assay was performed according to Bobo‐García et al. (2015). Briefly, 20 µL of filtered sample (0.45 µm, Sartorius Stedim) was added to 180 µL of 150 µM DPPH solution prepared in 80% methanol‐water solution. The mixture was incubated in the dark at room temperature for 40 min, and absorbance was measured at 515 nm using a UVM 340 microplate reader (Biochrom, Cambridge, England). The control was prepared using water instead of samples, and the results are expressed as a percentage of radical scavenging activity (RSA, %), calculated according to Equation 1.

(1) $$\mathrm{Radical}\;\mathrm{scavenging}\;\mathrm{activity}\;\left(\%\right)=\frac{\mathrm{Ac}-\mathrm{As}}{\mathrm{Ac}}\times 100$$

Where Ac is the absorbance of control and As is the absorbance of kombucha samples.

The ABTS assay was assessed following Re et al. (1999), with some adaptations. An aqueous solution of ABTS (7.0 mM) was mixed with potassium persulfate (140 mM) in a 5:1 ratio and incubated in the dark for 16 h. The solution was diluted with ethanol to an absorbance of 0.7 at 734 nm. For analysis, 20 µL of filtered sample (0.45 µm, Sartorius Stedim) was added to 280 µL of the diluted ABTS solution. After 6 min in low light conditions, the absorbance was measured at 734 nm in a UVM 340 microplate reader (Biochrom, Cambridge, England). ABTS^+^ solution was used as a control and the results were expressed as a percentage of radical scavenging activity (%), according to Equation 1.

### 2.7 Statistical Analysis

Experiments were conducted in triplicate, and the results are presented as mean ± standard deviation (SD). One‐way analysis of variance (ANOVA) and Tukey's test were performed to compare group means at a 5% significance level (p ≤ 0.05) using Prism 8.0 software (GraphPad Software, San Diego, CA, USA).

## 3 Results and Discussion

### 3.1 Powder Characterization

Moisture content and water activity are important properties for the stability of dried material, as low moisture and water activity (Aw) values contribute to slowing and/or avoiding deteriorating enzymatic reactions and microbial contamination (Moraes et al. 2024). All dried starter cultures showed moisture content and water activity values (Table 1) within safe limits to prevent microbial contamination and deterioration reactions, with values below 5% and 0.6, respectively (Ravichandran et al. 2023). Mohsin et al. (2022) found similar results for spray‐dried kombucha using gum Arabic as a carrier at 10% w/v, reporting 3.48 ± 0.13% of moisture content, a higher value than found in this study. Starter cultures obtained by spray drying (GA‐SD and MD‐SD) showed significantly lower moisture content when compared to freeze‐dried powders, which can be attributed to a more efficient drying process in SD compared to FD by involving faster evaporation rates at high temperature (Samborska et al. 2022).

**TABLE 1:** Physicochemical characterization of spray‐dried and freeze‐dried kombucha powders.

Sample Parameters	GA‐SD	GA‐FD	MD‐SD	MD‐FD
Moisture content (%)	2.31 ± 0.3^a^	3.11 ± 0.3^b^	2.22 ± 0.14^a^	3.62 ± 0.20^b^
Aw	0.135 ± 0.005^a^	0.210 ± 0.01^b^	0.172 ± 0.01^c^	0.217 ± 0.01^b^
pH	3.50 ± 0.1^a^	3.53 ± 0.3^a^	2.54 ± 0.2^b^	2.42 ± 0.2^b^
Hygroscopicity (g H_2_O/100 g)	10.0 ± 0.13^a^	9.80 ± 0.02^a^	8.32 ± 0.01^b^	7.21 ± 0.4^c^
Solubility (%)	95.30 ± 1.05^a^	94.50 ± 0.70^a^	93.90 ± 0.20^a^	92.90 ± 2.01^a^

*Note*: Results are presented as mean ± SD (n = 3). Different letters (a, b, c) indicate significant different groups (Tukey's test, p < 0.05). GA‐SD (fermented kombucha with gum Arabic, spray dried); GA‐FD (fermented kombucha with gum Arabic, freeze dried); MD‐SD (fermented kombucha with maltodextrin, spray dried); MD‐FD (fermented kombucha with maltodextrin, freeze dried).

Abbreviations: Aw, water activity.

Hygroscopicity refers to the material's ability to absorb moisture from the surrounding environment, and it is an important quality parameter, as hygroscopic powders are prone to caking and degradation over storage, decreasing their overall stability (Laureanti et al. 2023). Starter culture powders obtained with gum Arabic as a drying carrier showed significantly higher hygroscopicity (p < 0.05) (Table 1) than those prepared with maltodextrin. This difference can be associated with the chemical structure of gum Arabic, which presents higher water affinity due to the presence of hydroxyl groups (Al‐Hamayda et al. 2023). Moreover, spray‐dried samples showed higher hygroscopicity when compared with their respective freeze‐dried counterparts (p < 0.05). These results may be attributed to the lower moisture content observed in spray‐dried MD‐SD and GA‐SD samples (Rezende et al. 2018), which promotes a higher water concentration gradient between the dried material and the environment, resulting in a greater driving force for water adsorption (Sarabandi et al. 2019).

Solubility is one of the most important parameters that influence the consumer acceptability of dried food powders, being a key driver for several different applications (Ueda et al. 2023). All dried starter culture samples showed very high solubility, exceeding 90%, with no significant differences among experimental groups (p > 0.05), owing to the high solubility of the drying carriers used in this study (Moraes et al. 2024). The results found in this study are higher than those reported by Phan Van et al. (2024), which showed solubility ranging around 65–87% for microencapsulated kombucha cultures prepared through spray drying and freeze drying using maltodextrin and a blend of maltodextrin and gum Arabic. As for pH, GA‐based powders showed significantly higher values, which might be related to the more complex structure of gum Arabic, including glycoproteins, that may contribute to greater buffering capacity (Prasad et al. 2022).

**FIGURE 2:**
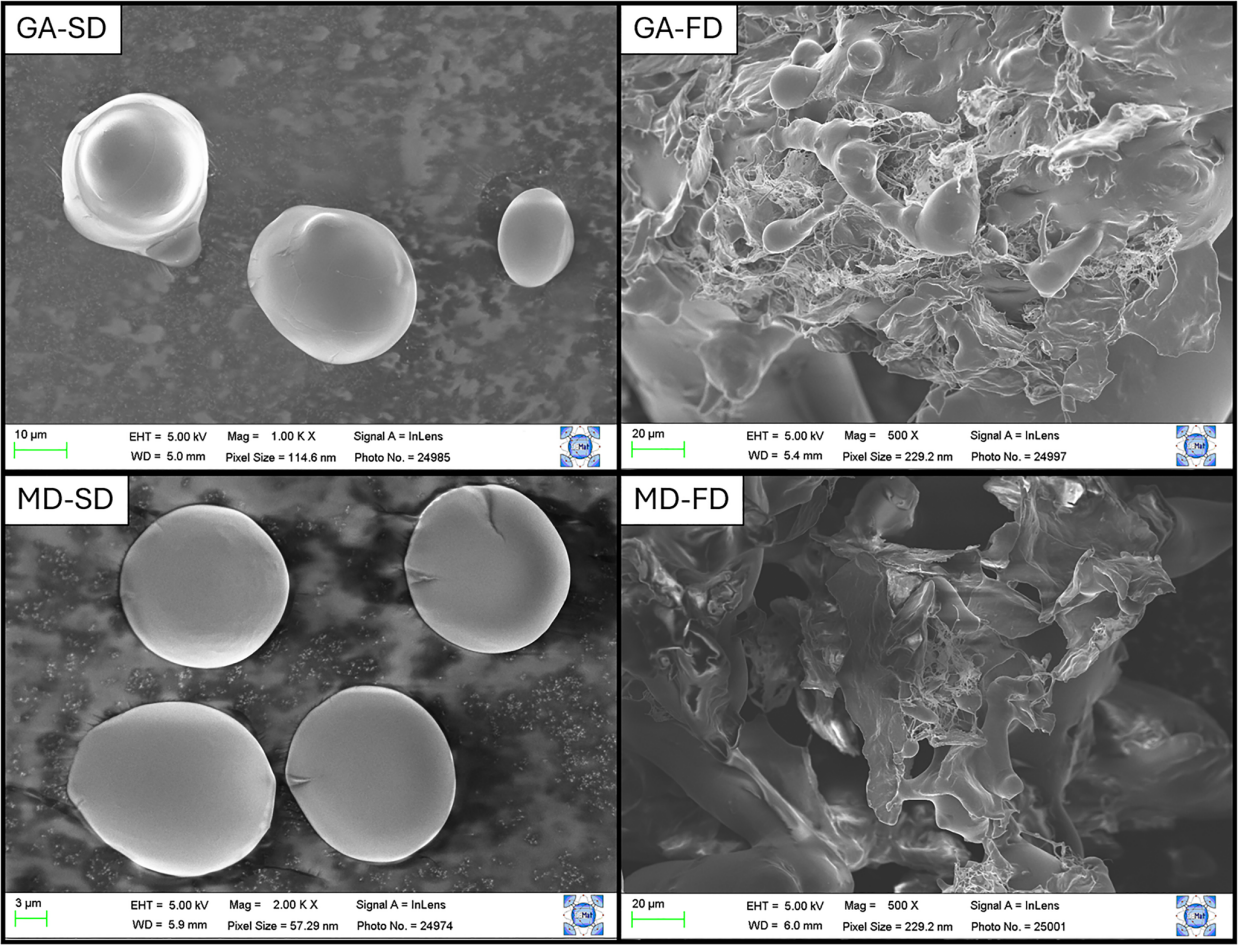
Scanning electron micrograph images of dried kombucha samples. Sample identification: GA‐SD (fermented kombucha with gum Arabic, spray dried); GA‐FD (fermented kombucha with gum Arabic, freeze dried); MD‐SD (fermented kombucha with maltodextrin, spray dried); MD‐FD (fermented kombucha with maltodextrin, freeze dried).

The morphology of dried kombucha starter cultures is presented in Figure 2. Particle shape may influence powder shelf stability, as rough and/or irregular particles have a bigger surface area exposed to oxygen, which might increase the rate of oxidation reactions. Moreover, rough particles are more prone to cracks in their outermost structure, which can expose the encapsulated core, compromising their stability (Ravichandran et al. 2023). Spray‐dried particles (GA‐SD and MD‐SD) showed smooth surfaces and spherical structure, which is expected for atomized particles, especially when prepared with highly soluble carriers with film‐forming abilities, such as GA and MD (Laureanti et al. 2023). Freeze‐dried samples showed a more porous and brittle structure, likely due to the preservation of the original matrix combined with shrinkage and aggregate formation during the process, which contribute to greater porosity and surface irregularity (Rezende et al. 2018). Similar behavior was observed by Misra et al. (2023) in probiotic‐GABA microcapsules produced by spray and freeze‐drying techniques.

**FIGURE 3:**
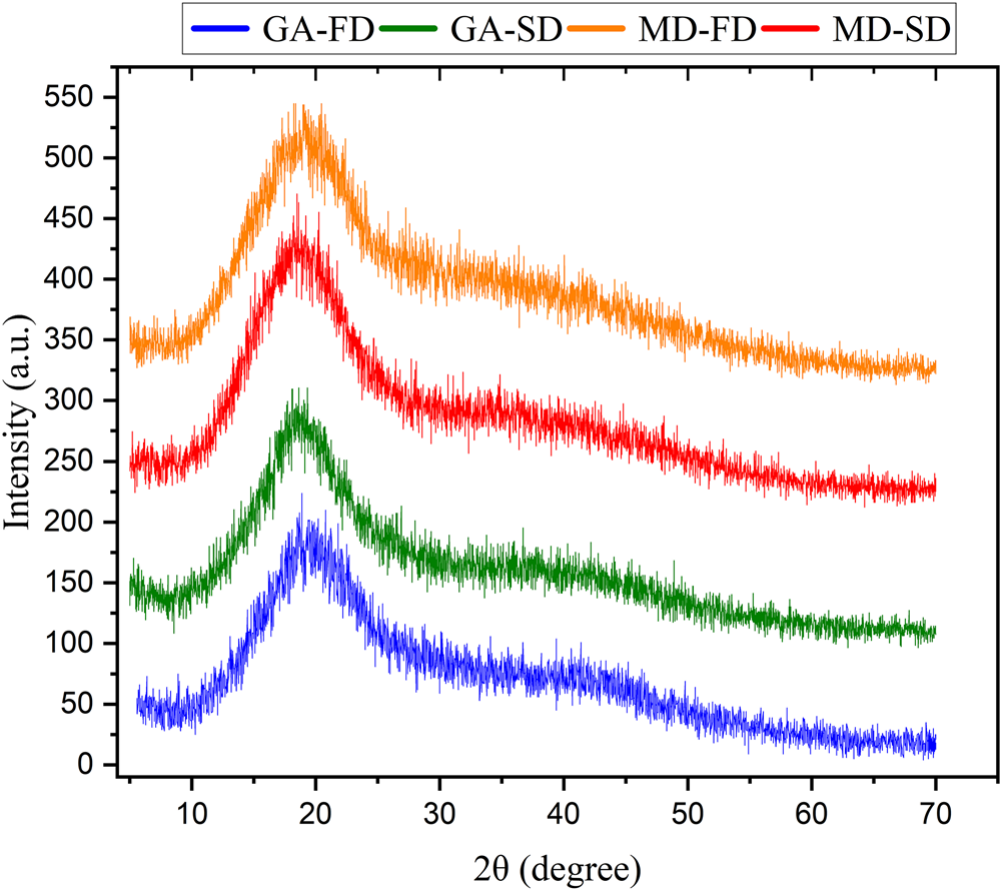
XRD patterns of dried kombucha samples. Sample identification: GA‐SD (fermented kombucha with gum Arabic, spray dried); GA‐FD (fermented kombucha with gum Arabic, freeze dried); MD‐SD (fermented kombucha with maltodextrin, spray dried); MD‐FD (fermented kombucha with maltodextrin, freeze dried).

The crystallinity of food powders is directly related to stability during storage. Highly crystalline materials are associated with lower water absorption, contributing to increased stability. Broad and diffuse peaks indicate amorphous structures, while well‐defined and sharp peaks indicate crystalline materials with a highly organized molecular structure (Moraes et al. 2024; Ravichandran et al. 2023). XRD spectra of the dried kombucha starter cultures (Figure 3) were similar across all experimental groups, suggesting that the different drying methods applied did not impact the microstructure of the samples. The predominance of diffuse peaks indicates that the powders have a largely amorphous structure, though a relatively more distinct peak was observed around 2θ = 20°. Similar results were obtained by Phan Van et al. (2024), who reported similar XRD patterns for kombucha inoculum powders produced via spray drying and freeze drying, showing a predominantly amorphous structure in microcapsules made with maltodextrin and a blend of maltodextrin and gum Arabic.

### 3.2 Evaluation of Kombucha Fermentation From Dried Starter Cultures

#### 3.2.1 Acetic Acid and Ethanol Production

**FIGURE 4:**
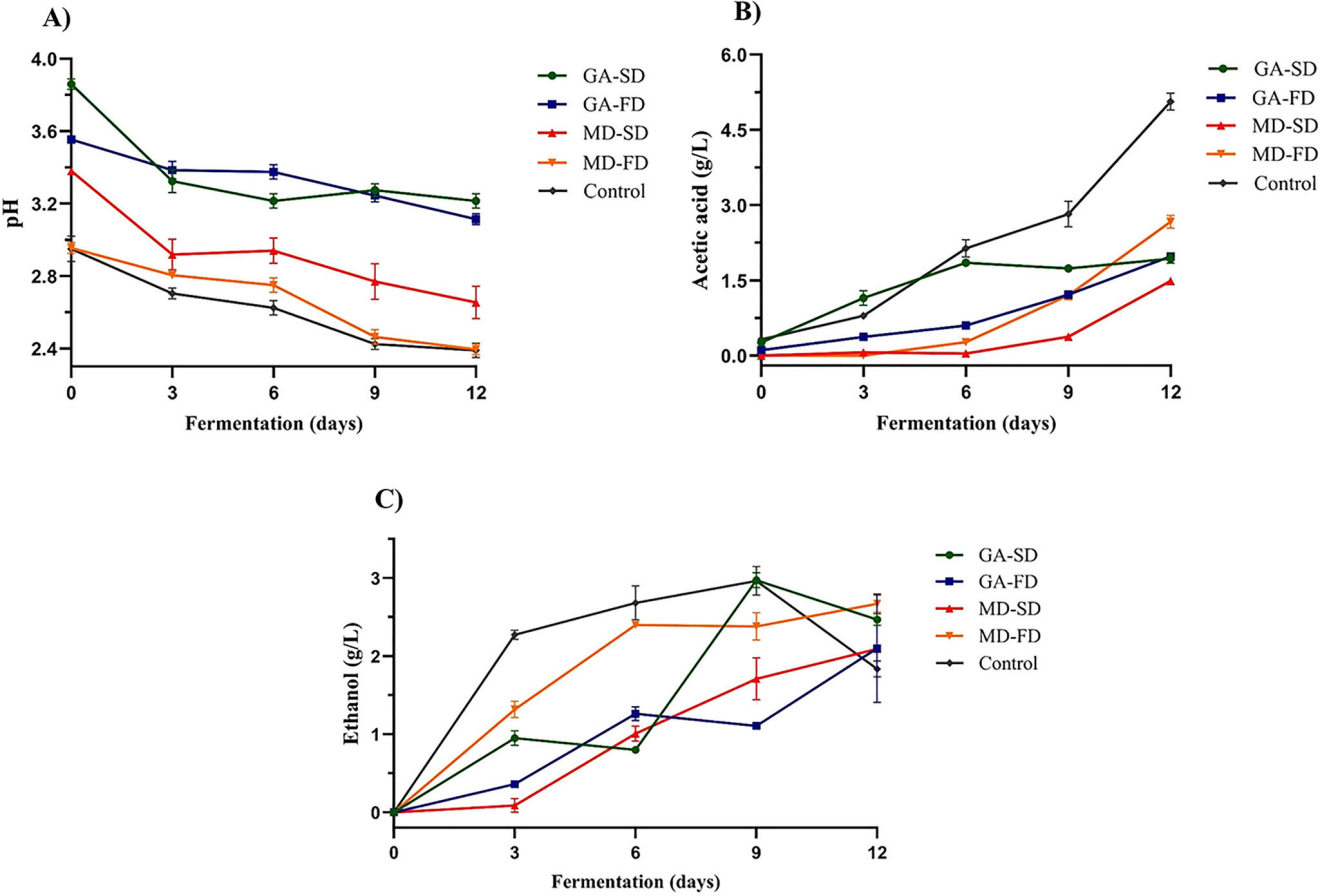
Fermentation metabolites of kombucha produced using dried starter cultures. (A) pH, (B) acetic acid production (g/L), and (C) ethanol production (g/L) during kombucha fermentation. Data are presented as means ± SD (n = 3). Sample identification: GA‐SD (fermented kombucha with gum Arabic, spray dried); GA‐FD (fermented kombucha with gum Arabic, freeze dried); MD‐SD (fermented kombucha with maltodextrin, spray dried); MD‐FD (fermented kombucha with maltodextrin, freeze dried); Control: Fresh kombucha liquid starter culture, not subjected to drying.

Dried starter cultures obtained through spray drying and freeze drying were used to produce fermented kombucha from fresh sweetened green tea infusions. Kombucha fermentation from dried starter cultures was carried out over the course of 12 days, during which a significant reduction of the pH of green tea infusion was observed (Figure 4A), primarily due to the production of organic acids, which are natural products from the SCOBY fermentation. Among these, acetic acid is one of the primary organic acids found in kombucha fermentation, produced via ethanol oxidation by the enzymatic activity of acetic acid bacteria (Savary et al. 2021; Wang et al. 2023), and its final concentration of acetic acid may vary according to the desired final sensory profile (Dartora et al. 2023).

The initial pH (day 0) was lower in kombucha with freeze‐dried starter cultures than in spray‐dried ones, indicating reduced metabolic activity after spray drying, consistent with the lower viable cell counts observed on day 0 (Table 2). After 12 days of fermentation, the pH of kombucha samples prepared from dried starter cultures ranged from pH 2.39 to 3.86 (Figure 4A), which is within the safe range to inhibit the growth of pathogenic microorganisms (pH < 4) (Cardoso et al. 2020). Samples prepared with GA‐derived dried starter cultures showed higher pH compared to control fermentation (prepared with commercial kombucha inoculum) and to samples prepared with MD‐derived dried starter culture (p < 0.05). This behavior might be related to the complex structure of gum Arabic, which includes glycoproteins in its composition (Prasad et al. 2022), potentially enhancing the buffering capacity and contributing to the higher pH observed during fermentation.

**TABLE 2:** Viable Cell Count of kombucha samples collected on days 0, 6, and 12 of fermentation.

Samples	Viable cell count (Log CFU/mL)								
	Day 0			Day 6			Day 12		
	AAB	YC	TC	AAB	YC	TC	AAB	YC	TC
GA‐SD	4.04 ± 0,06^a^	3.50 ± 0.03^a^	3.24 ± 0.09^a^	6.62 ± 0.16 ^ab^	6.47 ± 0.01^a^	6.65 ± 0.02^a^	6.86 ± 0.02^a^	6.76 ± 0.03^a^	6.59 ± 0.02^a^
GA‐FD	7.26 ± 0,0^b^	7.09 ± 0.01^b^	7.24 ± 0.02^b^	6.70 ± 0.1^b^	7.02 ± 0.03^b^	7.05 ± 0.04^b^	6.93 ± 0.04^a^	7.11 ± 0.06^b^	7.15 ± 0.01^c^
MD‐SD	3.32 ± 0.36^a^	3.33 ± 0.37^a^	3.36 ± 0.08^a^	6.95 ± 0.2^a^	6.85 ± 0.1^c^	6.61± 0.01^a^	6.31 ± 0.01^b^	5.48 ± 0.02^c^	5.56 ± 0.07^b^
MD‐FD	4.69 ± 0.12^c^	4.74 ± 0.17^d^	4.74 ± 0.46^d^	5.93 ± 0.04^d^	6.98 ± 0.04^b^	7.05 ± 0.06^b^	7.02± 0.03^c^	7.08 ± 0.05^b^	7.13 ± 0.13^c^
Control	5.28 ± 0.03^d^	5.22 ± 0.02^d^	5.41 0.11^d^	6.88 ± 0.21^a^	6.88 ± 0.07^abc^	6.88 ± 0.23^ab^	7.13 ± 0.07^d^	7.06 ± 0.02^b^	7.19 ± 0.1^c^

*Note*: Results are presented as mean ± SD (n = 3). Different letters (a, b, c, d) within the same column denote statistically significant differences between samples for the same microorganism group on the same day (Tukey's test, p < 0.05). GA‐SD (fermented kombucha with gum Arabic, spray dried); GA‐FD (fermented kombucha with gum Arabic, freeze dried); MD‐SD (fermented kombucha with maltodextrin, spray dried); MD‐FD (fermented kombucha with maltodextrin, freeze dried); Control: Fresh kombucha liquid starter culture, not subjected to drying.

Abbreviations: AAB, acetic acid bacteria count; YC, yeast count; TC, total aerobic plate count.

Acetic acid concentration ranged from 1.49 to 2.67 g/L for the samples prepared with dried starter cultures, at 12 days of kombucha fermentation. Considering the significant variability of the kombucha microbial consortium and the non‐standardized kombucha fermentation process, the concentrations found in this study are within the range presented in the literature, which shows great variability. Cardoso et al. (2020) reported acetic acid concentration close to 3 g/L in green tea kombucha after 10 days of fermentation, while Dartora et al. (2023) reported 7.61 ± 0.43 g/L of acetic acid after 14 days of fermentation in green tea kombucha. Acetic acid production in kombucha samples obtained from dried starter cultures was significantly lower than in the control fermentation with a liquid inoculum (5.10 ± 0.17 g/L) (Figure 4B), which points to an impact on the inoculum fermentation kinetics resulting from the use of a dried starter culture. Similar findings were shown by Fabricio et al. (2022), who reported a decrease in acetic acid production in kombucha fermented with a freeze‐dried microbial consortium (3.57 ± 0.14 g/L) compared to the control (5.56 ± 0.01 g/L).

Kombucha obtained from the MD‐SD starter culture showed the lowest acetic acid concentration at the end of fermentation, which shows that the spray‐drying process using maltodextrin as a carrier compromised the viability of acetic acid bacteria. Heat stress can affect membrane cell components and affects membrane structure, which can lead to cell inactivation (Cebrián et al. 2017). In this regard, non‐thermal freeze‐drying preserves the material structure and increases the maintenance of microbial cell viability (Li et al. 2024; Stefanello et al. 2019). Recent studies by Phan Van et al. (2024) have demonstrated the effectiveness of freeze‐drying compared to spray drying in preserving the viability and metabolic activity of kombucha starter cultures.

For kombucha fermentations with GA‐derived starter cultures, a similar pattern in pH reduction and acetic acid production is observed throughout fermentation, and no significant differences (p > 0.05) were observed between GA‐SD and GA‐FD samples at the end of fermentation. This result suggests that gum Arabic was effective as protective agent during spray drying process, possibly due to the higher film‐forming capacity, which might have led to attenuation of the adverse effects of spray drying (Al‐Hamayda et al. 2023). While previous studies have highlighted the protective properties of gum Arabic blends in bioactive compounds and microbial cells (Laureanti et al. 2023; Nguyen et al. 2022; Phan Van et al. 2024), the application of pure gum Arabic as a carrier for kombucha microbial consortium remains relatively understudied. Phan Van et al. (2024) reported no significant differences in acetic acid production when gum Arabic was incorporated into maltodextrin blends during the drying of kombucha starter culture.

The symbiotic activity of yeast and acetic acid bacteria is determinant for the final sensory profile of kombucha beverages (Tran et al. 2020). Yeasts in the kombucha consortium hydrolyze sucrose into monosaccharides and produce ethanol via anaerobic pathways, which is later converted into acetic acid (Meng et al. 2024). To comply with non‐alcoholic beverage regulations, which often have a maximum ethanol limit of 0.5% (v/v), low ethanol final concentrations are desirable in kombucha fermentation (De Miranda et al. 2022). Literature reports on ethanol concentrations in kombucha show significant variability, influenced by factors such as fermentation conditions, microbial composition, and substrate type, ranging from as low as 0.10% to as high as 3.56% ABV (Bishop et al. 2022; Jang et al. 2021), which is a challenge in accurately labeling kombucha as non‐alcoholic. Figure 4C shows ethanol production below the non‐alcoholic beverage limit after 12 days of fermentation, ranging from 2.1 to 2.7 g/L, with no significant differences among experimental groups and control (p > 0.05). Differently, Fabricio et al. (2022) observed a significant reduction in ethanol concentration (4.39 ± 0.09 g/L) in kombucha fermented with a freeze‐dried microbial community compared to the control (10.77 ± 0.07 g/L). The authors attributed this reduction to the absence of *Zymomonas* species, known for their high ethanol production capacity, in the freeze‐dried kombucha, whereas *Zymomonas* accounted for 53.62% of the microbial community in the control.

#### 3.2.2 Microbiological Characterization

The intricate interplay between yeast and bacteria drives the formation of the biochemical and sensorial characteristics of kombucha (Chou et al. 2024; Kim et al. 2023). The microbial consortium is also responsible for the biotransformation of phenolic compounds, directly contributing to the antioxidant activity of kombucha beverage (Liang et al. 2024; Shi et al. 2023). Assessing viable cell counts provides valuable insights into how drying processes may impact the metabolic activity of the microbial community, influencing the production of key metabolites and the bioactive properties of the resulting kombucha.

Table 2 presents the viable cell count of kombucha samples during fermentation. At 0 h, no significant differences were observed between carriers in spray‐dried samples for AAB, YC, and TC. In contrast, in freeze‐dried samples, GA‐FD showed significantly higher viable counts than MD‐FD, indicating a better protective effect against freeze‐drying stress. Additionally, freeze‐dried samples presented higher cell viability than spray‐dried ones, reinforcing the negative impact of heat stress on microbial survival. The lowest viable counts at day 0 were found for MD‐SD, which is in line with observations related to lower acetic acid production in kombucha samples fermented with that dried starter culture. Phan Van et al. (2024), who investigated the survival rates of *K. saccharivorans*, *S. cerevisiae*, and *L. brevis* in kombucha strains after spray‐drying and freeze‐drying, observed significant differences between the drying methods. Spray‐drying resulted in lower survival rates, particularly for *K. saccharivorans* and *S. cerevisiae*, while *L. brevis* exhibited greater resistance to spray‐drying and higher survival rates.

At day 12 of fermentation, kombucha fermentations carried out with dried starter cultures exhibited viable cell counts ranging from 6.31 to 7.02 log CFU/mL for acetic acid bacteria, 5.48 to 7.11 log CFU/mL for yeasts, and 5.56 to 7.15 log CFU/mL for total aerobic mesophilic bacteria. These values align with the typical range of 5 to 7 log CFU/mL commonly reported in the literature for viable cell counts in kombucha. Cardoso et al. (2020) observed microbial counts (acetic, lactic, and mesophilic bacteria, and yeasts) in both green and black tea kombucha fermented for 10 days, ranging from approximately 5 to 6 log CFU/mL. Neffe‐Skocińska et al. (2017) reported counts of acetic acid bacteria and yeasts reaching 7 log CFU/mL in kombucha fermented for 10 days. Additionally, it is observed that at the end of fermentation, the difference in viable cell counts compared to the control remains within a 1‐log range, except for MD‐SD, showing a difference of more than 1 log compared to the control. These findings show that despite the lower initial counts, dried starter cultures show feasible fermentation efficiency and are able to reach desirable viable counts within the standard fermentation time frame.

#### 3.2.3 TPC and Antioxidant Activity

Green tea infusions are a rich source of phenolic compounds, primarily flavonoids and phenolic acids (Musial et al. 2020). During kombucha fermentation, the microbial consortium is also responsible for the biotransformation of phenolic compounds, directly contributing to the antioxidant activity of kombucha beverage (Liang et al. 2024). Shi et al. (2023) demonstrated that kombucha microbial species, including *Acetobacter pasteurianus, Zygosaccharomyces bailii*, and *Debaryomyces hansenii*, enhanced the levels of epigallocatechin gallate, an abundant tea polyphenol, while *Z. bailii* and *Acetobacter xylinum* showed an increase in tea polyphenols with low molecular weight.

**FIGURE 5:**
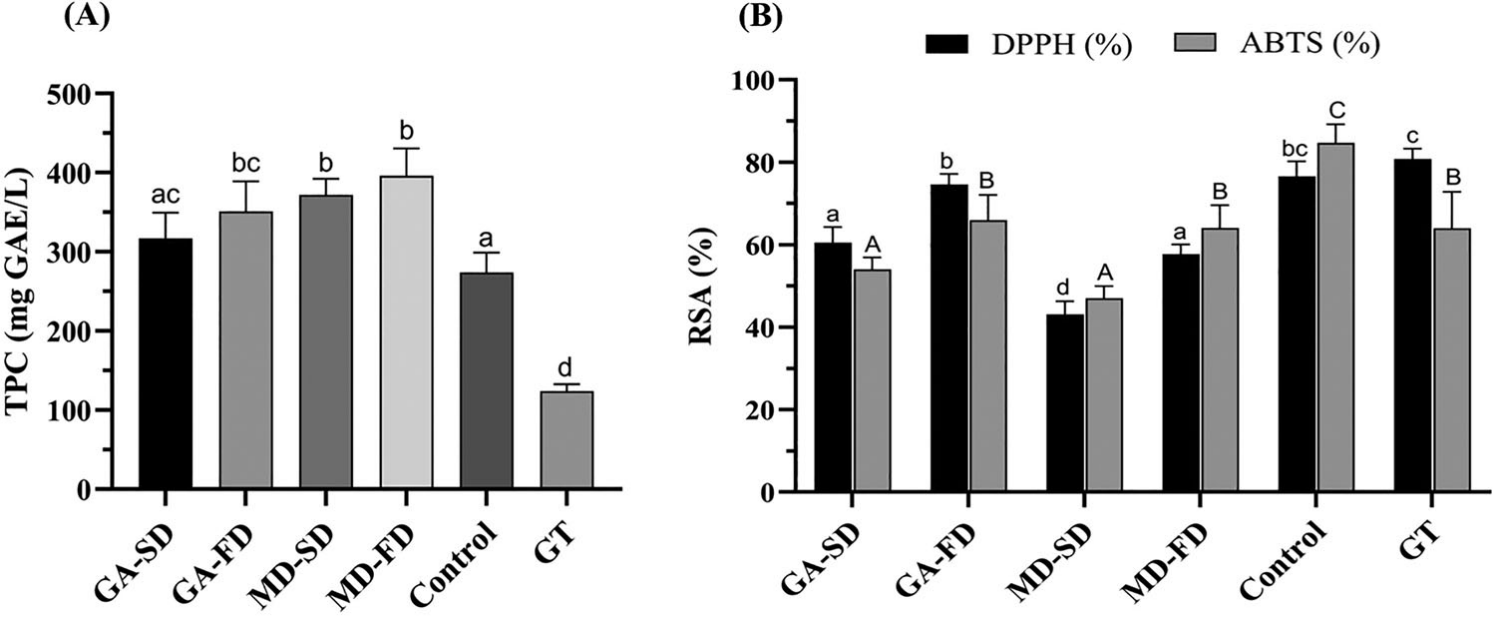
(A) Total phenolic compounds and (B) Radical scavenging activity (RSA) of kombucha samples after 12 days of fermentation. Different letters (a, b, c, d) indicate significant differences (Tukey's test, p < 0.05). Lowercase letters refer to DPPH results (%) and uppercase letters refer to ABTS (%). Sample identification: GA‐SD (fermented kombucha with gum Arabic, spray dried); GA‐FD (fermented kombucha with gum Arabic, freeze dried); MD‐SD (fermented kombucha with maltodextrin, spray dried); MD‐FD (fermented kombucha with maltodextrin, freeze dried); Control: Fresh kombucha liquid starter culture, not subjected to drying; GT: Green tea infusion.

TPC in kombucha presents considerable variability. For green tea kombucha, Dartora et al. (2023) observed 377.17 ± 6.36 mg GAE/L after 14 days, while Jakubczyk et al. (2020) found 320.10 ± 3.50 mg GAE/L after the same fermentation time. TPC in fermented kombucha samples ranged from 271.32 to 391.47 mg GAE/L (Figure 5A). Although some variation was observed, no significant differences were found between samples produced with the same carrier agent, regardless of the drying method used. Except for GA‐SD, all kombucha samples obtained with dried starter cultures exhibited significantly higher TPC compared to the control kombucha produced with a commercial liquid inoculum. This effect may be partially attributed to the encapsulation of polyphenols present in the kombucha feed suspension used for drying. The higher TPC values also suggest that the microbial consortium retained its metabolic activity after drying, as evidenced by increased phenolic content compared to the green tea infusion. This is relevant since microbial metabolism plays a central role in the biotransformation of phenolic compounds, contributing to the functional quality of the final beverage (Liang et al. 2024).

Similarly, Phan Van et al. (2024) showed TPC ranging from 199.90 to 344.26 mg GAE/L in black tea kombucha produced using spray‐dried and freeze‐dried kombucha starters, with higher values in kombucha fermented with freeze‐dried starters compared to spray‐dried. Additionally, when comparing the TPC values of green tea infusion and the fermented kombucha samples, a positive impact of fermentation on total phenolics is observed, which is consistently supported by literature reports (Aung and Eun 2021; Kilic and Sengun 2023; Liang et al. 2024; Shi et al. 2023).

Kombucha is widely recognized for its functional potential, particularly its antioxidant capacity, responsible for radical scavenging activity against reactive oxygen species, inhibiting oxidative reactions (Muscolo et al. 2024).

Antioxidant activity of kombucha is mainly associated with the presence of bioactive compounds, especially polyphenols and flavonoids (Jakubczyk et al. 2020; Oliveira et al. 2023). Differently from TPC, the antioxidant activity measured by DPPH and ABTS radical scavenging activities in the kombucha sample obtained from the control inoculum was higher than those samples obtained with dried starter cultures (Figure 5B). Although TPC is often associated with antioxidant capacity, this correlation is not always linear due to differences in phenolic composition, stability, and bioavailability (Kim et al. 2023). In this study, some samples with higher TPC showed lower DPPH activity, which may be attributed to the specific phenolic profile or matrix interactions that limit radical scavenging potential. Additionally, microbial composition plays a key role in modulating antioxidant capacity through the bioconversion of phenolic compounds. Kim et al. (2023) demonstrated that antioxidant potential varies with microbial combinations, emphasizing the influence of bacterial and yeast strains. Nonetheless, as also reported by Fabricio et al. (2022), antioxidant activity in kombuchas from dried starter cultures was generally lower than that of the control, which was associated with alteration in microbial symbiosis after freeze‐drying.

DPPH radical scavenging activity ranged from 43.1% to 74.6% for kombucha samples obtained with dried starter cultures, with MD‐SD exhibiting the lowest value (43.1% ± 3.15) and GA‐FD presenting the highest value (74.6% ± 2.57), with no significant difference from the control sample (76.6%) (p > 0.05). ABTS radical scavenging activity ranged from 46.9 to 66%, with the control presenting the highest antioxidant activity (85% ± 4.48). Jakubczyk et al. (2020) observed an 88.23 ± 0.83% DPPH radical scavenging activity in green tea kombucha after 14 days of fermentation, a value comparable to that reported by Silva et al. (2021) (86.9%) for green tea kombucha fermented for the same duration. These values are also similar to the DPPH radical scavenging activity observed in the control sample in this study. Kilic and Sengun (2023) reported a high ABTS+ radical scavenging activity exceeding 90% inhibition in black tea kombucha samples, and Chou et al. (2024) observed a significant ABTS antioxidant capacity of 78.75% in black tea kombucha fermented for 10 days.

## 4 Conclusion

This study demonstrates the feasibility of using spray drying and freeze drying to produce stable kombucha starter cultures, offering a potential solution for microbial preservation and process standardization. While freeze‐drying better preserved microbial viability, gum Arabic proved effective in mitigating the thermal stress of spray‐drying. Despite differences in fermentation performance, all dried cultures successfully initiated kombucha fermentation, maintaining key metabolic and functional properties. Due to its industrial scalability, the GA‐SD formulation emerges as a promising strategy for large‐scale production of kombucha starter cultures. These findings highlight the potential for scalable, shelf‐stable kombucha starter cultures that can enhance industrial production, ensuring consistency and reducing reliance on fresh inoculum. Future research should focus on optimizing drying parameters, identifying the microbial species, and characterizing the phenolic profile of kombucha throughout fermentation to better understand the biochemical transformations involved and their impact on product functionality.

## Author Contributions

**Alliny Samara Lopes de Lima**: conceptualization, writing – original draft, formal analysis, writing – review and editing, methodology, software. **Ana Terra de Medeiros Felipe**: writing – review and editing, data curation, investigation, validation. **Maria Eduarda de Souza da Cruz**: formal analysis, methodology, investigation. **Luiz da Silva Ferreira Junior**: investigation, methodology, formal analysis. **Francisco Canindé de Sousa Junior**: project administration, resources, data curation, writing – review and editing, visualization. **Fábio Gonçalves Macêdo de Medeiros**: writing – review and editing, data curation, validation, investigation, visualization. **Márcia Regina da Silva Pedrini**: validation, data curation, supervision, resources, project administration, funding acquisition, conceptualization, visualization.

## Conflicts of Interest

The authors declare no conflicts of interest.

## Data Availability

Data will be made available on request.
